# Gut-Sourced Vasoactive Intestinal Polypeptide Induced by the Activation of α7 Nicotinic Acetylcholine Receptor Substantially Contributes to the Anti-inflammatory Effect of Sinomenine in Collagen-Induced Arthritis

**DOI:** 10.3389/fphar.2018.00675

**Published:** 2018-06-26

**Authors:** MengFan Yue, XinYu Zhang, YanNong Dou, ZhiFeng Wei, Yu Tao, YuFeng Xia, Yue Dai

**Affiliations:** ^1^Department of Pharmacology of Chinese Materia Medica, China Pharmaceutical University, Nanjing, China; ^2^Experiment Center of Teaching and Learning, Shanghai University of Traditional Chinese Medicine, Shanghai, China

**Keywords:** sinomenine, rheumatoid arthritis, cholinergic anti-inflammatory pathway, α7 nicotinic acetylcholine receptor, vasoactive intestinal polypeptide

## Abstract

Sinomenine has long been used for the treatment of rheumatoid arthritis in China. However, its anti-inflammatory mechanism is still debatable because the *in vitro* minimal effective concentration (≥250 μM) is hardly reached in either synovium or serum after oral administration at a therapeutic dose. Recent findings suggest that the α7 nicotinic acetylcholine receptor (α7nAChR) might mediate the inhibitory effect of sinomenine on macrophage activation, which attracts us to explore the anti-arthritis mechanism of sinomenine by taking neuroendocrine-inflammation axis into consideration. Here, we showed that orally administered sinomenine ameliorated the systemic inflammation of collagen-induced arthritis (CIA) rats, which was significantly diminished by either vagotomy or the antagonists of nicotinic acetylcholine receptors (especially the antagonist of α7nAChR), but not by the antagonists of muscarinic receptor. Sinomenine might bind to α7nAChR through interacting with the residues Tyr184 and Tyr191 in the pocket. In addition, the generation of vasoactive intestinal polypeptide (VIP) from the gut of CIA rats and cultured neuron-like cells was selectively enhanced by sinomenine through the activation of α7nAChR-PI3K/Akt/mTOR pathway. The elevated levels of VIP in the serum and small intestine of rats were negatively correlated with the scores of joint destruction. The crucial role of VIP in the anti-arthritic effect of sinomenine was confirmed by using VIP hybrid, a non-specific antagonist of VIP receptor. Taken together, intestine-sourced VIP mediates the anti-arthritic effect of sinomenine, which is generated by the activation of α7nAChR.

**GRAPHICAL ABSTRACT:**
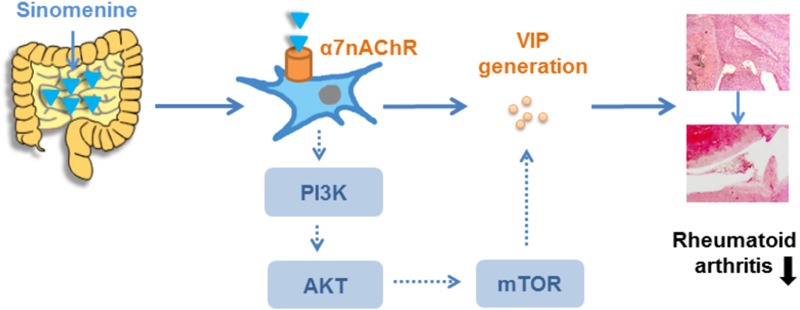
Sinomenine exerts anti-arthritic effect through gut-sourced VIP via activation of α7nAChR.

## Introduction

Rheumatoid arthritis, one of the most prevalent chronic inflammatory joint diseases, can cause cartilage/bone damage and disability, and has developed to be a substantial burden for both the individuals and the society (Smolen et al., 2016). SIN, an alkaloid derived from the root and stem of *Sinomenium acutum*, was previously demonstrated to have substantial anti-inflammatory, immunosuppressive, and anti-arthritic properties (Sun et al., 2014), and has long been used as a first line drug for RA treatments in China. Several research groups attempted to illustrate the anti-arthritic mechanisms of SIN by *in vitro* studies, and showed that SIN could suppress osteoclast formation and fibroblast-like synoviocyte activation at concentrations over 250 μM (Chen et al., 2011; Li et al., 2013). Unfortunately, our recent pharmacokinetics study demonstrated that oral administration of SIN (120 mg/kg) for consecutive 14 days showed a relative lower plasma concentration in CIA rats. The plasma concentration in CIA rats peaked at only about 9 μM (Tong et al., 2015). With regard to the pharmacokinetics–pharmacodynamics disconnection, we offered a point that the anti-arthritic effect of oral SIN might be gut-dependent (Tong et al., 2016), and the precise mechanism needs to be identified.

The recent discovery that nerve system takes part in regulating inflammatory response has made the CAP a novel therapeutic target for inflammatory diseases. CAP is mainly composed of vagus nerve, neurotransmitter ACh and its receptors. Vagus nerve is a mixed nerve in the autonomic nervous system containing afferent fibers that convey sensations and efferent fibers that leads to the release of ACh. ACh released from synaptic junctions or other cells under vagus nerve control binds to and activates its receptors classified as muscarinic ACh receptor (M receptor) and nicotinic ACh receptor (N receptor) to exert physiological functions. Although the exact mechanism how CAP modulates systemic and peripheral inflammation remains unclear, the activation of ACh receptors is generally acknowledged to be an integral step. In several animal models, such as colitis and arthritis (Wang et al., 2004; van Maanen et al., 2009; Hayashi et al., 2014), cholinergic activation by electric stimulation of vagus nerve or by administration of cholinergic agonists was shown to inhibit the release of inflammatory cytokines and attenuate inflammation. What is noteworthy is that the vagus nerve innervates most part of the digestive tract, and modulates gastro-intestinal motility and secretion as well as maintains the homeostasis of infection and inflammation in the gut. According to these important roles of vagus nerve in gut, it is possible that the gut-dependent anti-inflammatory action of SIN may be related to the stimulation on CAP.

Accumulating evidences suggest that neuropeptides, widely distributing in the central and peripheral nervous systems, participate in the modulation of inflammatory responses (Ben-Horin and Chowers, 2008). In autoimmune disorders, some neuropeptides such as VIP, SST, and CCK have been proven to play important roles in regulating the balance between pro-inflammatory and anti-inflammatory responses. For example, CCK treatment markedly hindered the occurrence and development of mouse CIA, and reduced the production of pro-inflammatory cytokines and chemokines in the joints and cultured synovial cells (Li et al., 2011). Thus, neuropeptides might function as a link between vagus nerve stimulation and inflammation modulation.

In the present study, we verified the participation of vagus nerve in the anti-arthritic effect of SIN, and explored the gut-relevant anti-inflammatory mechanism of SIN from a perspective of CAP-neuropeptide modulation. Importantly, we provided evidence that anti-inflammatory neuropeptide originating from the peripheral nervous system acts as a crucial modulator of the systemic inflammation in RA.

## Materials and Methods

### Reagents

Sinomenine (purity > 98%) was purchased from Nanjing Zelang Pharmaceutical Technology Co., Ltd. (Nanjing, China). NIC was purchased from Chengdu Must Bio-Technology Co., Ltd. (Chengdu, China). PILO nitrate injection was purchased from Wuxi Xingzhou Medicine Co., Ltd. (Wuxi, China). HEX chloride, rapamycin and chicken CII were purchased from Sigma-Aldrich (St. Louis, MO, United States). Complete Freund’s adjuvant was obtained from Becton Drive Co., Ltd. (Franklin Lakes, NJ, United States). α-BTX was purchased from Shanghai Boyao Biological Technology Co., Ltd. (Shanghai, China). VIP hybrid was purchased from Wuxi Asia Peptide Biological Technology Co., Ltd. (Wuxi, China). DMEM/F12 medium and TRIzol reagent were purchased from Invitrogen (Carlsbad, CA, United States). Rat TNF-α, IL-6, IL-1β, IFN-γ, IL-17A, TGF-β, and IL-10 ELISA kits were purchased from Dakewe Biotech Co., Ltd. (Shenzhen, China). Rat VIP ELISA kit was purchased from Shanghai Guangrui Biological Technology Co., Ltd. (Shanghai, China). HiScript^TM^ Q RT SuperMix and AceQ^TM^ qPCR SYBR^®^ Green Master Mix were purchased from Vazyme Biotech Co., Ltd. (Piscataway, NJ, United States). H-89, GF109203X, U0126, and LY294002 were purchased from Beyotime Company (Haimen, China). Antibodies against p-Akt, p-RPS6, total Akt and total RPS6 were purchased from Sangon Biotech, Co., Ltd. (Shanghai, China). Other chemicals and reagents used were of analytical grade.

### Animals

Female Wistar rats weighting 130–150 g were obtained from the Comparative Medicine Center of Yangzhou University (Yangzhou, China). The animal experiments were conducted with the approval of the Animal Ethics Committee of China Pharmaceutical University, and conformed to the National Institute of Health guidelines on the ethical use of animals. Animals were housed under a 12 h light/dark cycle (22 ± 2°C), and fed with a standard chow diet and water.

### Vagotomy

Cervical vagotomy or sham surgery was performed 4 days before the arthritis induction as previously described (Kanashiro et al., 2016). In brief, rats were anesthetized with 4% chloral hydrate hydrochloride, and performed a cervical midline incision to expose and transect the left cervical vagus trunk. In the sham-operated rats, the left cervical vagus nerve was exposed but not transected.

### Collagen-Induced Arthritis and Treatment

Collagen-induced arthritis was established in rats with reference to the method described previously (Kokkola et al., 2003). Briefly, CII was emulsified in complete Freund’s adjuvant (1 mg/mL). CII emulsion was intradermally injected at the base of the rat tail at a volume of 0.2 mL. Seven days later, a booster immunization was conducted at the base of the rat tail with a volume of 0.1 mL CII, avoiding the primary injection sites. The day of first immunization was recorded as day 0. Rats were observed daily for clinical signs of arthritis, and each paw was scored on a scale of 0–4: 0 = no swelling or erythema, 1 = slight swelling and or erythema, 2 = low to moderate edema, 3 = pronounced edema with limited joint usage, and 4 = excess edema with joint rigidity. The total score for each rat was calculated as an arthritis index with a maximum value of 16. The volumes of hind paws were determined by a paw volume plethysmometer.

On day 14, rats were divided into different groups according to the articular index scores and paw volumes, and administered with SIN (120 mg/kg, i.g.), PILO (2 mg/kg, i.p.), or NIC (0.3 mg/kg, i.p.) from day 14 to day 28. The co-administration of ATR (1 mg/kg, i.p.), HEX (3.5 mg/kg, i.p.), α-BTX (1 μg/kg, i.p.) or VIP hybrid (0.1 mg/kg, i.p.) was daily conducted 30 min before SIN administration.

### Histopathological Examination

Rats were euthanized with an excess dose of chloral hydrate hydrochloride on day 28. The ankle joints were taken and fixed in 10% neutral buffered formalin for 48 h, decalcified with EDTA, embedded in paraffin, and sliced into 5 mm-thick sections. HE staining was performed, and histologic changes of joints were determined by a pathologist blinded to the experimental groups.

### Cell Culture

Rat pheochromocytoma cells (PC12 cells) were obtained from Chinese Center for Type Culture Collection (Wuhan, China), and maintained in DMEM/F12 medium supplemented with 10% fetal calf serum, penicillin (100 U/mL) and streptomycin (100 U/mL) at 37°C under a humidified 5% CO_2_ atmosphere. PC12 cells were exposed to recombinant mouse nerve growth factor (50 ng/mL) for 7 days to induce neuron-like differentiation, and seeded on 6-well plates (5 × 10^5^ cells/well) for the following experiments. After pretreated with or without α-BTX (0.1 μM), U0126 (10 μM), LY-294002 (10 μM), H-89 (10 μM), GF109203X (10 μM), or rapamycin (10 nM) for 30 min, cells were treated with SIN (0.03, 0.1, or 0.3 mM) for 24 h. The supernatant were collected for ELISA, and the cells were collected for western blot assay.

### ELISA

Cytokine and VIP levels in the cell supernatant and serum were measured using ELISA kits according to the manufacturer’s instructions. The operation steps are as follows: (1) add samples and biotinylated antibodies into precoated wells, close plates with closure plate membranes, and incubate at 37°C for 60–120 min; (2) uncover closure plate membrane, discard liquid, add washing buffer to each well, still for 60 s then drain, repeat three times, and dry by pat; (3) add streptavidin-HRP to each well, close plates with closure plate membranes, and incubate at 37°C for 30–60 min; (4) repeat step 2; (5) add TMB to each well, evade the light and preserve for 5–15 min at 37°C, stop the reaction with stop solution.

### Quantitative PCR Assay

Total RNAs from cultured cells, small intestines, or synovium tissues of rats were extracted with TRIzol reagent, and reverse transcribed to cDNA using iScript cDNA Synthesis kit. Real-time PCR assay was performed with Bio-Rad MyiQ2 detection System using AceQ^TM^ qPCR SYBR^®^ Green Master Mix. The primer sequences used were listed in **Table 1**. The expressions of genes were normalized to β-actin, and the results were evaluated using comparative threshold cycle (2^-ΔΔC_t_^) method.

**Table 1:** Primers used in quantitative real-time PCR.

Target genes		Sequence (5′→3′)
CCK	Forward	GGTCCGCAAAGCTCCCTC
	Reverse	CCGAAATCCATCCAGCCCAT
Foxp3	Forward	AAAAGGAGAAGCTGGGAGCTATG
	Reverse	GCTACGATGCAGCAAGAGCTCT
IL-10	Forward	CCCTCTGGATACAGCTGCG
	Reverse	GCTCCACTGCCTTGCTTTTATT
IL-17A	Forward	GCCGAGGCCAATAACTTTCT
	Reverse	GAGTCCAGGGTGAAGTGGAA
RORγT	Forward	TGCCTTCCTTCTTCCTAGTTGA
	Reverse	TGCCAGTGATGTCCTTCTCC
SP	Forward	CGCAATGCAGAACTACGAAAGA
	Reverse	CGCGGACACAGATGGAGAT
SST	Forward	GACCCCAGACTCCGTCAGTT
	Reverse	GGCATCGTTCTCTGTCTGGTT
VIP	Forward	CCAGAAGCAAGCCTCAGTTCCT
	Reverse	GCAGCCTGTCATCCAACCTCAC
β-actin	Forward	TGCCTCTGGTCGTACCACTG
	Reverse	TCTCTTCGACACGATACAACGG

### Western Blot

The total cell lysates from PC12 cells were prepared by suspending cells in NP40 buffer (Beyotime, Nanjing, China). Samples were separated by SDS-PAGE and transferred to NC membranes. The membranes were then blocked with 5% non-fat milk for 2 h, incubated with specific primary antibodies at 4°C overnight, incubated with IRDye-conjugated secondary antibody at 37°C for 1 h, and detected by using Odyssey Infrared Imaging System (LI-COR, Inc., Lincoln, MT, United States).

### Cell Viability Assay

The viability of PC12 cells was determined using the 3-(4,5-dimethyl-2-thiazolyl)-2,5-diphenyl-2H-tetrazolium bromide (MTT) assay. Cells were seeded in 96-well plates, and treated with SIN for 24 h. 20 μL of MTT solution (5 mg/mL) was added to each well 4 h before the end of incubation. Then, the supernatant was removed, and the formazan crystal was dissolved with 150 μL of DMSO. After 10 min, the optical absorbance at wavelength of 570 nm was read with a Model 1500 Multiskan spectrum microplate Reader (Thermo, Waltham, MA, United States).

### Molecular Docking

To investigate the possible binding mode of SIN to α7nAChR, a three-dimensional structure was generated from the molecular connection tables in the molecular operating environment. Template compounds were downloaded from Protein Data Bank (Protein Data Bank code: 3SQ6). According to the GPCR alignment constraints, the sequence alignment was adjusted. Using molecular docking technique, the best model was obtained by GBVI/WSA dG score. Energy minimized and hydrogens were added using Protonate 3D in the molecular operating environment to remove over twisted compound conformation.

### Statistical Analysis

Data of *in vivo* and *in vitro* experiments were presented as the means ± SEM. Comparisons between multiple groups were performed using one-way analysis of variance (ANOVA) and *post hoc* Tukey’s test. *P*-values less than 0.05 were considered to be statistically significant.

## Results

### The Integrity of Vagus Nerve Was Essential for the Anti-arthritic Effect of Sinomenine

**FIGURE 1:**
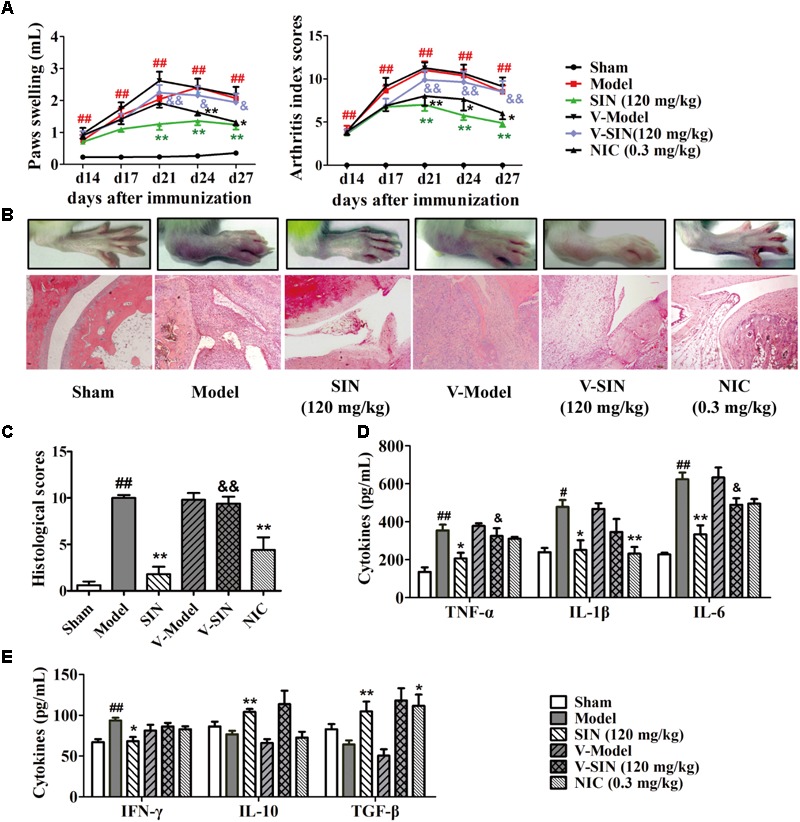
Influence of vagotomy on the anti-arthritic effect of sinomenine (SIN). Rats were subjected to left cervical vagotomy or sham surgery 4 days before the induction of CIA. SIN was intragastrically administered daily for 2 weeks from day 14 to day 28. Nicotine (NIC), as positive control, was intraperitoneally injected from day 14 to 28. Rats in other groups were administered with an equal volume of vehicle during the treatment period. **(A)** The paw swelling and arthritis index were determined. **(B)** Photographs of representative hind paws were taken, and histopathological changes of right ankle joints in each group were determined by HE staining (original magnification 200×). **(C)** The histological scores of each group were calculated. **(D)** The serum levels of TNF-α, IL-1β, and IL-6 were measured by ELISA. **(E)** The serum levels of IFN-γ, IL-10, and TGF-β were measured by ELISA. Data were expressed as means ± SEM, *n* = 6–8. *F*-values were 18.37 for paw swelling and 10.32 for arthritis index in **(A)**. ^#^*P* < 0.05 and ^##^*P* < 0.01 versus Sham; ^∗^*P* < 0.05 and ^∗∗^*P* < 0.01 versus Model; ^&^*P* < 0.05 and ^&&^*P* < 0.01 versus SIN.

To elucidate the role that vagus nerve plays in the anti-arthritic effect of SIN, we performed left cervical vagotomy or sham surgery 4 days before the induction of rat CIA. No sickness behavior or mortality occurred in the several following weeks. At day 14 after the first immunization, rats subjected to either left cervical vagotomy or sham surgery started to show paw swelling and erythema. The symptoms grew severe in the next 7 days and did not show significant self-recovery till the end of the experiment. Cervical vagotomy had no significantly influence on the severity of CIA. Daily administration of SIN (120 mg/kg, i.g.) markedly attenuated the disease progression, reduced the disease severity, decreased the histopathological scores of ankle joints, and down-regulated the serum levels of pro-inflammatory cytokines (namely TNF-α, IL-1β, and IL-6) in CIA rats subjected to sham surgery. In unilateral vagotomized CIA rats, however, SIN failed to alleviate paw swelling and to reduce the serum levels of pro-inflammatory cytokines (**Figures 1A–D**). In addition, SIN significantly down-regulated the serum level of IFN-γ, and up-regulated the serum levels of IL-10 and TGF-β in CIA rats. However, cervical vagotomy did not significantly influence the regulatory effect of SIN on the T cells-related cytokines, including IFN-γ, IL-10, and TGF-β (**Figure 1E**). These results suggested that vagus nerve integrity was essential for the anti-inflammatory effect of SIN in CIA rats.

### The Antagonist of N Receptor but Not M Receptor Reversed the Anti-arthritic Effect of Sinomenine in CIA Rats

**FIGURE 2:**
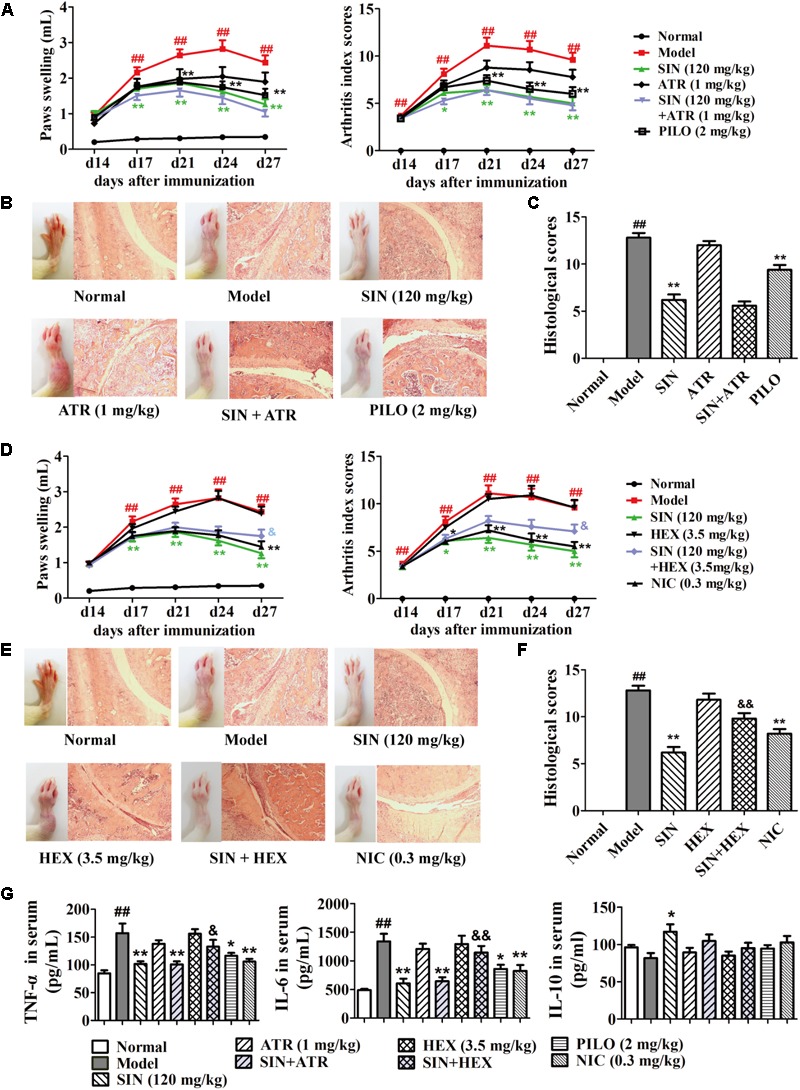
Influence of M and N receptor antagonists on the anti-arthritic effect of sinomenine (SIN). Rats were intragastrically administered with SIN and intraperitoneally injected with ATR, pilocarpine (PILO), hexamethonium (HEX) or nicotine (NIC) from day 14 to day 28. (All data in this figure are from the same group of animal.) ATR or HEX was given 30 min before the administration of vehicle or SIN. **(A,D)** The paw swelling and arthritis index were determined. **(B,E)** Photographs of representative hind paws were taken, and histopathological changes of right ankle joints in each group were determined by HE staining (original magnification 200×). **(C,F)** The histological scores of each group were calculated. **(G)** The serum levels of TNF-α, IL-6, and IL-10 were measured by ELISA. Data were expressed as means ± SEM, *n* = 6–8. *F*-values were 3.92 for paw swelling and 6.59 for arthritis index in **(A)**, and *F*-values were 11.98 for paw swelling and 9.25 for arthritis index in **(D)**. ^#^*P* < 0.05 and ^##^*P* < 0.01 versus Normal; ^∗^*P* < 0.05 and ^∗∗^*P* < 0.01 versus Model; ^&^*P* < 0.05 and ^&&^*P* < 0.01 versus SIN.

To identify the subtypes of ACh receptor involved in the anti-arthritic effect of SIN, we employed the antagonists of both M receptor (ATR) and N receptor (HEX) in the *in vivo* experiments, and the relative agonists were used as positive control. Data showed that although the agonists of both M receptor (PILO) and N receptor (NIC) significantly ameliorated rat CIA, including alleviation of paw swelling and joint damage as well as reduction of the serum levels of TNF-α and IL-6, only HEX significantly diminished the anti-arthritic effect of SIN (**Figures 2A–F**). Of note, HEX did not affect the role of SIN in up-regulating the serum level of IL-10, which is an anti-inflammatory cytokine (**Figure 2G**).

### The Antagonist of α7nAChR Reversed the Anti-arthritic Effect of Sinomenine in CIA Rats

**FIGURE 3:**
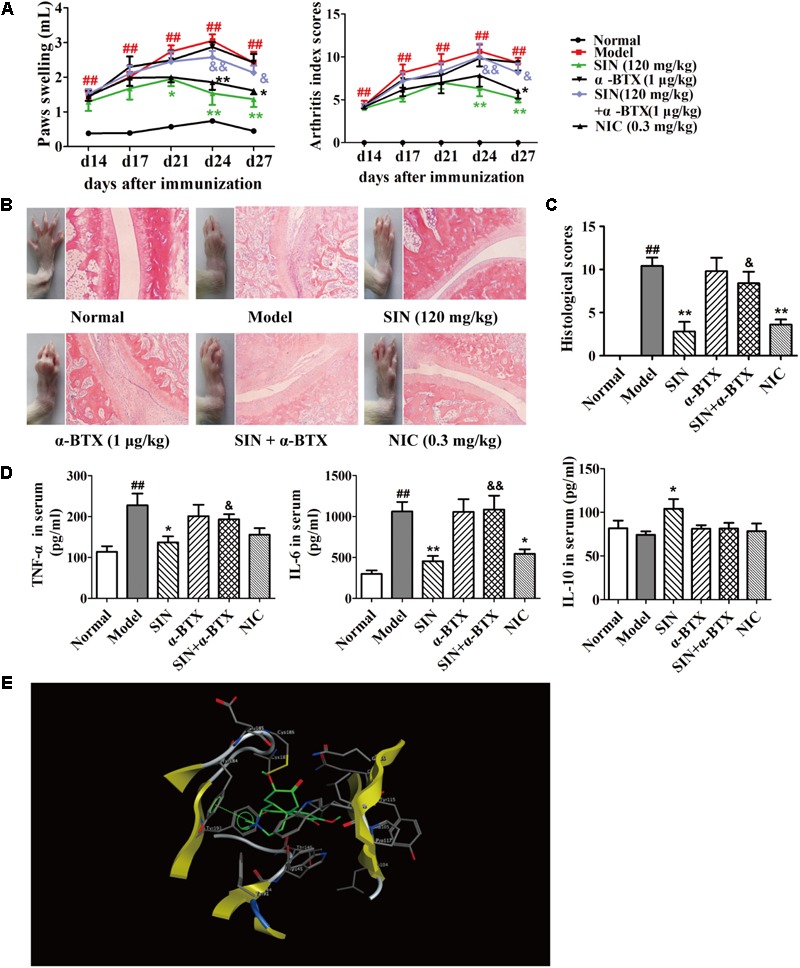
Influence of α7nAChR antagonist on the anti-arthritic effect of sinomenine (SIN). Rats were intragastrically administered with SIN and intraperitoneally administered with α-bungarotoxin (α-BTX) or nicotine (NIC) from day 14 to day 28. α-BTX was given 30 min before the administration of vehicle or SIN. **(A)** The paw swelling and arthritis indexes were determined. **(B)** Photographs of representative hind paws were taken, and histopathological changes of right ankle joints in each group were determined by HE staining (original magnification 200×). **(C)** The histological scores of each group were calculated. **(D)** The serum levels of TNF-α, IL-6, and IL-10 were measured by ELISA. **(E)** The theoretical possibility of SIN binding to α7nAChR was illustrated by molecular docking. Data were expressed as means ± SEM, *n* = 6–8. *F*-values were 13.02 for paw swelling and 8.67 for arthritis index in **(A)**. ^#^*P* < 0.05 and ^##^*P* < 0.01 versus Normal; ^∗^*P* < 0.05 and ^∗∗^*P* < 0.01 versus Model; ^&^*P* < 0.05 and ^&&^*P* < 0.01 versus SIN.

Among the family of N receptors, α-BTX-sensitive nicotinic receptor especially α7nAChR has been suggested to play an important role in modulating inflammation response via activating the CAP (Wang et al., 2003). We next examined whether α7nAChR was indispensable for the anti-arthritic effect of SIN. Our data showed that after co-administered with α-BTX (1 μg/kg), the anti-arthritic effect of SIN (120 mg/kg) was almost disappeared. The rats co-administrated with α-BTX and SIN exhibited serious arthritic symptom and high levels of pro-inflammatory cytokines. This result was similar as that observed in the rats of CIA model group, implying that α7nAChR played a key role in the anti-inflammatory effect of SIN (**Figures 3A–D**). To identify whether SIN can directly interact with α7nAChR, we established a molecular docking model and observed the interactions between SIN and the residues in the pocket of α7nAChR. The result showed that SIN was theoretically possible to interact with Tyr18 and Tyr191 among all the residues (**Figure 3E**).

### Sinomenine Selectively Induced the Generation of VIP in the Small Intestines of CIA Rats

**FIGURE 4:**
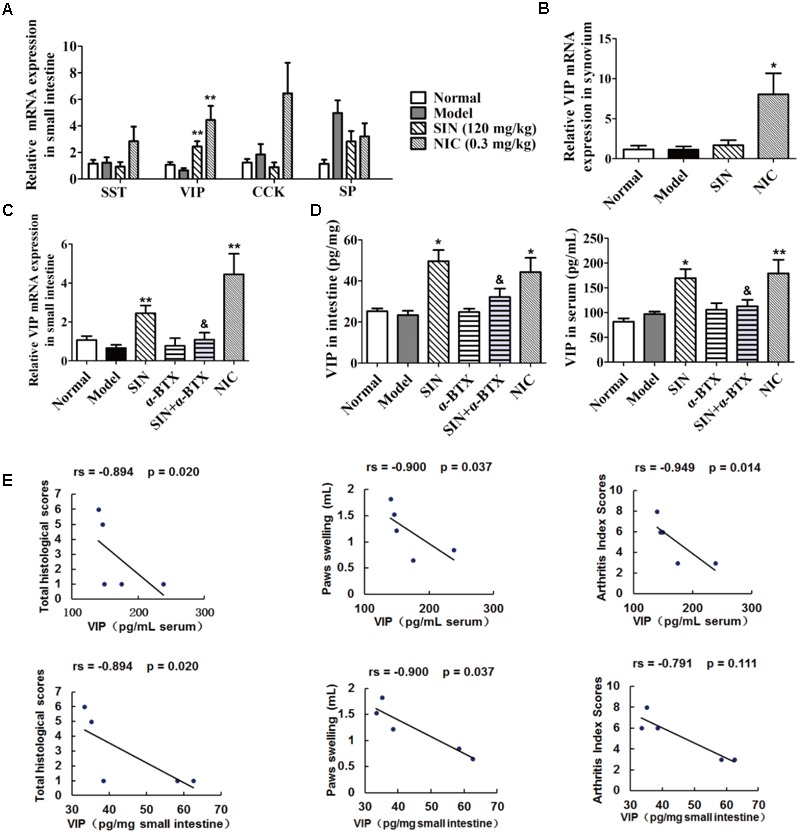
Influence of sinomenine (SIN) on neuropeptides in serum, small intestine and synovium of CIA rats. Rats were intragastrically administered with SIN and intraperitoneally administered with α-bungarotoxin (α-BTX) or nicotine (NIC) from day 14 to day 28. α-BTX was administered 30 min before the administration of vehicle or SIN. **(A)** The mRNA expressions of SST, VIP, CCK, and substance P (SP) in small intestine were measured by quantitative PCR assay. **(B)** The mRNA expression of VIP in synovium was measured by quantitative PCR assay. **(C)** The mRNA expression of VIP in small intestine was measured by quantitative PCR assay. **(D)** The level of VIP in small intestines and sera were determined by ELISA. **(E)** Correlations of intestinal/serum level of VIP with histological scores, paws swelling, or arthritis index scores were evaluated by spearman bivariate correlation analysis. Rs, Spearman’s rank correlation coefficient. *P*-values less than 0.05 were considered as significant difference. The relative expressions of genes measured by quantitative PCR assay were normalized to that of β-actin. Data were expressed as means ± SEM, *n* = 5. ^∗^*P* < 0.05 and ^∗∗^*P* < 0.01 versus Model; ^&^*P* < 0.05 versus SIN.

Mounting evidence suggests that neuropeptides might act as message senders between neuro-immune crosstalk in the gut. Our data showed that SIN treatment markedly enhanced the expression of VIP in the intestines of CIA rats, but did not affect the expression of other three neuropeptides namely SST, CCK, and substance P (**Figure 4A**). However, SIN was unable to enhance the expression of VIP in the synovium of CIA rats (**Figure 4B**). These results further confirmed our previous postulation that the pharmacological effect of SIN initially happens in the gut. NIC, an agonist of N receptor, significantly elevated the level of VIP in both gut and synovium (**Figures 4A,B**). A co-administration with α-BTX (1 μg/kg) markedly diminished the effect of SIN in up-regulating the VIP levels in intestine and serum of CIA rats (**Figures 4C,D**). This result implied that SIN induced the expression of VIP via activating α7nAChR. Furthermore, the intestinal and serum levels of VIP in SIN-treated rats was highly negatively correlated with the histological scores of the joints, the degree of paw swelling, and the arthritis scores (**Figure 4E**), suggesting that VIP might be the key mediator responsible for the anti-arthritic effect of SIN.

### Sinomenine Induced the Generation of VIP in Neuronal Cells

**FIGURE 5:**
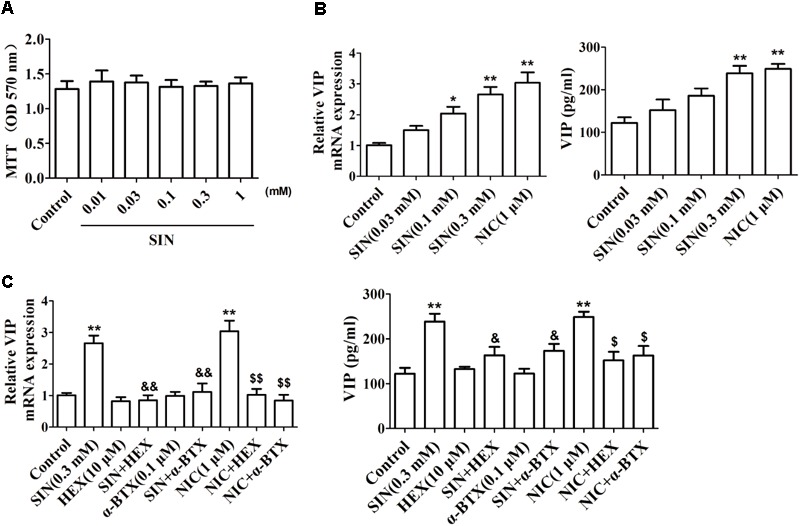
Effect of sinomenine (SIN) on the expression of VIP in PC-12 cells. **(A)** Effect of SIN on the cell viability of PC-12 cells was measured by MTT assay. **(B)** Effect of SIN and nicotine (NIC) on the mRNA expression and protein level of VIP in PC-12 cells were measured by quantitative PCR assay and ELISA respectively. **(C)** Effects of hexamethonium (HEX) and α-bungarotoxin (α-BTX) on the up-regulated VIP level by SIN and NIC in PC-12 cells were measured by quantitative PCR assay and ELISA respectively. Data were expressed as means ± SEM of three independent experiments. ^∗^*P* < 0.05 and ^∗∗^*P* < 0.01 versus Control; ^$^*P* < 0.05 and ^$$^*P* < 0.01 versus NIC; ^&^*P* < 0.05 and ^&&^*P* < 0.01 versus SIN.

To confirm the influence of SIN on VIP generation in peripheral nerves, PC12 cell line was cultured *in vitro* and treated with nerve growth factor (50 ng/mL) to differentiate into neuron-like cells. As shown in **Figure 5A**, SIN did not show obvious cytotoxicity at concentrations up to 1 mM, but concentration-dependently enhanced the mRNA expression of VIP in differentiated PC12 cells and elevated the VIP protein level in the cell supernatants. NIC (1 μM) treatment also enhanced VIP expression as compared with control (**Figure 5B**). Addition of either HEX (10 μM) or α-BTX (0.1 μM) largely diminished the expression of VIP induced by SIN and NIC (**Figure 5C**). It was therefore concluded that α7nAChR activation played a crucial role in the intestinal generation of VIP caused by SIN treatment.

### Sinomenine Induced the Generation of VIP Through PI3K/Akt/mTOR Pathway

**FIGURE 6:**
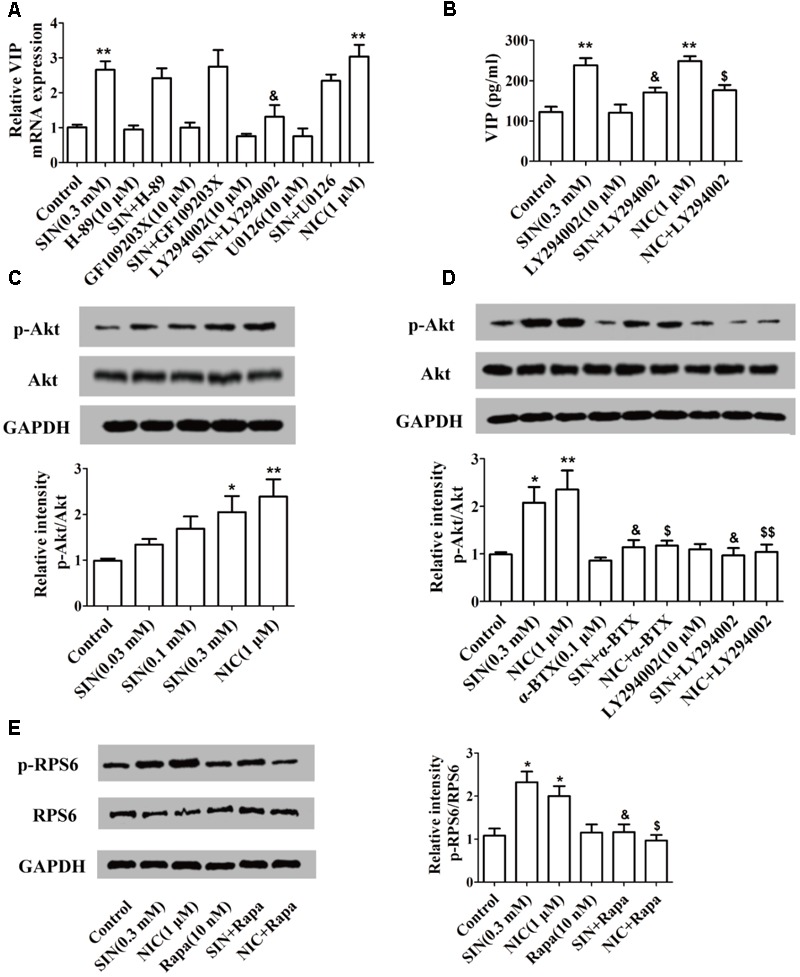
Involvement of α7nAChR and PI3K/Akt/mTOR pathway in the sinomenine (SIN)-induced VIP expression in PC-12 cells. **(A,B)** Cells were pre-treated with H-89, GF109203X, LY-294002 or U0126 (10 μM). After 30 min, cells were exposed to vehicle, nicotine (NIC) or SIN for 24 h. The mRNA and protein level of VIP were measured by quantitative PCR assay and ELISA respectively. **(C)** The expression of Akt and p-Akt were detected by western blot. **(D)** Cells were pre-treated with LY-294002 or α-bungarotoxin (α-BTX, 0.1 μM). After 30 min, they were exposed to vehicle, NIC (1 μM) or SIN (0.3 mM) for 24 h. The expression of Akt and p-Akt were detected by western blot. **(E)** Cells were pre-treated with rapamycin (Rapa, 10 nm). After 30 min, they were exposed to vehicle, NIC (1 μM) or SIN (0.3 mM) for 24 h. The expression of RPS6 and p-RPS6 were detected by western blot. Data were expressed as means ± SEM of three independent experiments. ^∗^*P* < 0.05 and ^∗∗^*P* < 0.01 versus Control; ^$^*P* < 0.05 and ^$$^*P* < 0.01 versus NIC; ^&^*P* < 0.05 versus SIN.

To recognize the signaling pathways by which SIN induced VIP expression, H-89 (a specific inhibitor of PKA), GF109203X (a specific inhibitor of PKC), LY294002 (a specific inhibitor of PI3K) and U0126 (a specific inhibitor of MEK1/2) were co-added with SIN and NIC. LY-294002 partially diminished the up-regulation of VIP mRNA expression caused by either SIN or NIC in PC12 cells, while H-89, GF109203X, or U0126 did not affect the role of SIN or NIC on VIP mRNA expression (**Figure 6A**). Consistently, we found that up-regulation of VIP protein level in the cell supernatant was also abolished by the inhibition of PI3K (**Figure 6B**). The phosphorylations of Akt and RPS6, two downstream molecules of PI3K, were measured in the cells treated with SIN (0.03, 0.1, 0.3 μM) or NIC (1 μM). SIN concentration-dependently induced the phosphorylations of Akt and RPS6. HEX (10 μM), α-BTX (0.1 μM), or LY-294002 (10 μM) diminished the phosphorylation of Akt induced by SIN and NIC. Rapamycin (10 nM) notably abolished the phosphorylation of RPS6 induced by SIN and NIC (**Figures 6C–E**). These results demonstrated that both SIN and NIC induced the expression of VIP via PI3K/Akt/mTOR signaling pathway

### The Antagonist of VIP Receptor Reversed the Anti-arthritic Effect of Sinomenine in CIA Rats

**FIGURE 7:**
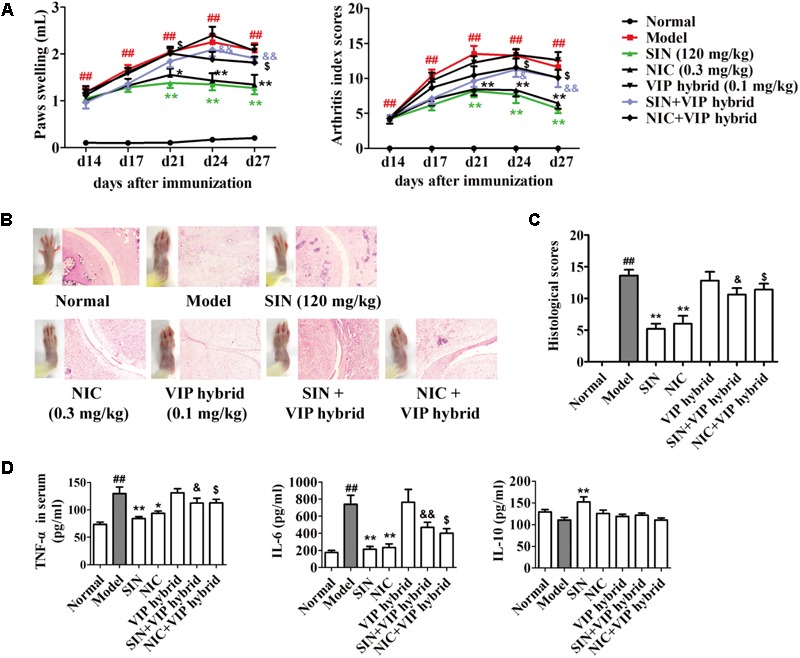
Influence of the antagonist of VIP receptor on the anti-arthritic effect of sinomenine (SIN). Rats were intragastrically administered with SIN and intraperitoneally administered with VIP hybrid or nicotine (NIC) from day 14 to day 28. VIP hybrid was administered 30 min before the administration of vehicle, NIC or SIN. **(A)** The paw swelling and arthritis index were determined. **(B)** Photographs of representative hind paws were taken, and histopathological changes of right ankle joints in each group were determined by HE staining (original magnification 200×). **(C)** The histological scores of each group were calculated. **(D)** The serum levels of TNF-α, IL-6, and IL-10 were measured by ELISA. Data were expressed as means ± SEM, *n* = 6–8. *F*-values were 9.86 for paw swelling and 11.45 for arthritis index in **(A)**. ^##^*P* < 0.01 versus Normal; ^∗^*P* < 0.05 and ^∗∗^*P* < 0.01 versus Model; ^$^*P* < 0.05 versus NIC; ^&^*P* < 0.05 and ^&&^*P* < 0.01 versus SIN.

As mentioned above, the spearman bivariate correlation analysis indicated that the anti-arthritic effect of SIN was highly correlated with its enhancement on VIP generation (**Figure 4D**). To further verify the correlation, VIP hybrid, a selective antagonist of VIP receptor, was co-administered with SIN in CIA rats. The results showed that VIP hybrid itself did not affect the pathological features and the serum levels of pro-inflammatory cytokines in CIA rats, but it markedly attenuated the anti-arthritic effect of SIN in views of paw swelling, AI scores and histopathological scores (**Figures 7A–C**), as well as the serum levels of pro-inflammatory cytokines (TNF-α and IL-6) (**Figure 7D**).

## Discussion

Alleviating inflammation has been recognized as an effective therapeutic strategy to reduce painful symptom and disease progression in RA patients. SIN, as an anti-arthritic medicine, has been recorded in the Chinese Pharmacopeia and clinically used for decades. It can ameliorate the local and systemic inflammatory responses in RA patients and experimental animals in spite of a very low plasma exposure after oral administration (Zhou et al., 2008; Tong et al., 2015). With regard to the pharmacokinetic–pharmacodynamics disconnection of SIN, the present study addressed its underlying mechanism in views of CAP and consequent generation of intestinal neuropeptides.

The CAP has recently been implicated as an important anti-inflammatory pathway in the amelioration of various inflammatory diseases, such as myocarditis, peritonitis, pancreatitis, and arthritis (van Maanen et al., 2009; Liu et al., 2010; Cheng et al., 2014; Ma et al., 2016). Vagotomy could increase the susceptibility to develop colitis in murine models by enhancing the production of pro-inflammatory cytokines and the expression of NF-κB in the lamina propria (Di Giovangiulio et al., 2016), but does not significantly affect the susceptibility to develop RA in human (Carlens et al., 2007). However, both vagus nerve electrical stimulation and activation of α7nAChR by NIC or selective agonists could reduce the levels pro-inflammatory cytokines and attenuated the pathological changes in experimental arthritis (van Maanen et al., 2009; Levine et al., 2014). In consistent with these findings, our present study showed that vagotomy itself did not affect the severity of arthritis in CIA rats, but it markedly attenuated the anti-arthritic effect of oral SIN. As vagus nerve is indispensable in CAP, we postulated that SIN ameliorates arthritis via activating CAP.

α7 nicotinic acetylcholine receptor, abundantly expressed on numerous cells in the central and peripheral nervous system as well as the immune system, plays a key role in the CAP. In various inflammatory diseases such as pancreatitis and multiple sclerosis, α7nAChR knockout increases disease severity, while treating with the α7nAChR agonists reduce the inflammation and ameliorate the diseases (Millet et al., 2017). In consistent with the previous report that SIN inhibited LPS-induced macrophage activation in an α7nAChR-related manner (Yi et al., 2015), our results showed that the antagonists of α7nAChR and non-selective N receptors blocked the anti-arthritic effect of SIN in CIA rats. In combination with our previous report that the anti-arthritic effect of SIN might be gut-dependent (Tong et al., 2015), the activation of intestinal α7nAChR might be pivotal for SIN to ameliorate arthritis.

Up until now, the exact mechanism by which CAP reduces the systemic and peripheral inflammation has not been clearly elucidated. Several reports suggest that the CAP produce anti-inflammatory functions through down-regulation of the number of macrophages in the synovium, inhibition of TNF-α and HMGB1 expression, and regulation of the T cells imbalance (Li et al., 2010, 2016; Wu et al., 2014). Although current studies on α7nAChR activation are mainly performed in macrophages rather than neurons, it is still controversial whether the ACh interacts with macrophages since the vagus nerve does not directly interact with macrophages anatomically. The vagus nerve preferentially interacts with enteric neurons that express neuropeptides, and the nerve endings of these neuropeptide producing neurons are located close to gut resident inflammatory cells (Bonaz et al., 2016). Our data showed that SIN markedly enhanced the expression of VIP in small intestines of CIA rats and in neuron-like cells. The antagonist of α7nAChR significantly inhibited the effect of SIN on VIP induction *in vitro* and *in vivo*. Given that the antagonist of VIP receptor largely diminished the anti-arthritic effect of SIN, it is possible that VIP acts as a message sender between CAP activation and arthritis attenuation.

Vasoactive intestinal polypeptide is a major endogenous neuropeptide abundantly distributed in the central and peripheral nervous systems. VIP can be synthesized and released by the parasympathetic nerve and by activated T cells (Ganea et al., 2015). As an immunoregulatory molecule, VIP was reported to inhibit the expression of a wide spectrum of pro-inflammatory cytokines such as TNF-α, IL-6, IL-12, and iNOS (Delgado et al., 2004). Treatment with VIP could decrease the incidence of and delay the onset of rat arthritis, and protect the bones from RANKL-induced resorption (Deng et al., 2010). In the present study, we found that NIC treatment up-regulated VIP expression in both CIA rats and neuron-like cells. In addition, SIN could activate α7nAChR and enhance the production of VIP. The findings suggested that VIP generation due to the activation of α7nAChR could be a crucial step in the function of CAP. SIN did not obviously affect the expression of VIP in synovial tissues of CIA rats, and the elevated level of VIP in the serum probably originated from the enteric nervous system. According these evidences, we concluded that SIN alleviated the inflammatory response in CIA rats through activating α7nAChR and consequently enhancing VIP generation from gut.

Multiple kinases such as PKA, PKC, PI3K, and MEK were reported to be implicated in the synthesis and release of VIP (Vaudry et al., 2002; Hook et al., 2008; Holighaus et al., 2011; de Heuvel et al., 2012). Based on our preliminary screening, only the inhibitor of PI3K could effectively interfere with the effect of SIN on the generation of VIP. Further studies demonstrated that the activation of downstream Akt and mTOR were also enhanced by the treatment of either SIN or NIC in an α7nAChR dependent manner. Overall, our current data suggest that the activation of α7nAChR and downstream PI3K/Akt/mTOR pathway was essential in SIN-induced VIP expression in neuron-like cells. Since the detailed mechanism for VIP synthesis remains unclear, further studies are needed to investigate the relationship between the activation of PI3K/Akt/mTOR pathway and the expression of VIP, especially in enteric neurons.

## Conclusion

The present study demonstrates that the anti-arthritic effect of SIN is dependent on the integrity of vagus nerve, and its anti-inflammatory mechanism can be summarized step-by-step as: stimulation of α7nAChR – activation of PI3K/Akt/mTOR pathway – generation of anti-inflammatory neuropeptide VIP from the nicotinic cholinergic nerves in small intestine – VIP enters systemic circulation to attenuate inflammatory response. These findings provide a new and plausible explanation for the anti-arthritic effect of oral SIN.

## Author Contributions

MY, XZ, and YuD designed the study. MY, XZ, YaD, and YT performed the experiments, YX, ZW, and YuD contributed reagents and technical suggestions. MY and YuD wrote the paper.

## Conflict of Interest Statement

The authors declare that the research was conducted in the absence of any commercial or financial relationships that could be construed as a potential conflict of interest. The reviewer AR and handling Editor declared their shared affiliation.

## Abbreviations

α-BTX: α-bungarotoxin
ACh: acetylcholine
ATR: atropine
CAP: cholinergic anti-inflammatory pathway
CCK: cholecystokinin
CIA: collagen-induced arthritis
CII: type II collagen
ELISA: enzyme linked immunosorbent assay
HE: hematoxylin and eosin
HEX: hexamethonium
NIC: nicotine
PILO: pilocarpine
RA: rheumatoid arthritis
SEM: standard errors of mean
SIN: sinomenine
SST: somatostatin
VIP: vasoactive intestinal polypeptide
